# On the identity of the fossil aquatic beetles from the Tertiary localities in the southern part of the Upper Rhine Graben (Coleoptera, Hydrophilidae, Dytiscidae)

**DOI:** 10.3897/zookeys.78.800

**Published:** 2011-01-28

**Authors:** Fikáček Martin, Jiří Hájek, Heiko Schmied

**Affiliations:** 1 Department of Entomology, National Museum, Kunratice 1, CZ-148 00 Praha 2, Czech Republic; 2 Department of Zoology, Faculty of Science, Charles University in Prague, Viničná 7, CZ-128 44 Praha 2, Czech Republic; 3 Steinmann Insitute of Geology, Mineralogy and Palaeontology, University of Bonn, Nußalle 8, 53115 Bonn, Germany; 4 Institute of Crop Science and Resource Conservation, Animal Ecology, University of Bonn, Melbweg 42, 53127 Bonn, Germany

**Keywords:** Dytiscidae, Hydrophilidae, *Copelatus*, *Hydrobius*, fossils, Eocene-Oligocene boundary, Brunstatt, Kleinkems, neotype designation

## Abstract

This study focuses on the fossil beetles assigned previously to the family Hydrophilidae described from the localities in the southern part of the Upper Rhine Graben: Brunstatt (France, Alsace) and Kleinkems (Germany, Baden-Württemberg) (both dated ca. to Eocene-Oligocene boundary, 34 Ma). The identity of Escheria convexa Förster, 1891 is fixed by the designation of its neotype, the species is redescribed, illustrated, transferred from the hydrophilid genus Hydrobius Leach, 1815 to the genus Copelatus Erichson, 1832 (Coleoptera: Adephaga: Dytiscidae) and compared with other fossil representatives of Copelatus. The identity of the remaining three species, Hydrobius crassipunctatus (Förster, 1891), Hydrobius dimidiatus (Förster, 1891) and Hydrobius punctulatus (Förster, 1891),is briefly evaluated on the basis of the original descriptions and illustrations only, because their types were lost or destroyed during World War II; all three species are removed from the fossil record of the Hydrophiloidea and placed into Polyphaga *incertae sedis*. The geology and stratigraphy of Brunnstatt and Kleinkems are discussed briefly.

## Introduction

In his study of the insects from the Tertiary outcrop of Brunstatt (Alsace, France), Förster (1891) described four species of the family Hydrophilidae, assigning all of them to the fossil genus Escheria Heer, 1847. Later, Théobald (1937) recorded two of these species from the locality of Kleinkems (spelled incorrectly as ‘Kleinkembs’ by the latter author), which is situated 16 km from Brunstatt and is believed to be of the same age and origin (see below for details). Based on the preserved characters, Théobald (1937) transferred all four of Förster’s (1891) species to the recent hydrophilid genus Hydrobius Leach, 1815. After 1937, the specimens were never re-examined and their identity remained unclear. Unfortunately, the Förster collection was either lost or destroyed during World War II (see Material and methods for details), which further complicates research.

For this study, we have re-examined the specimens from the locality of Kleinkems studied previously by Théobald (1937). In order to resolve the complicated situation concerning the four hydrophilid taxa, a neotype is designated for Escheria convexa Förster, 1891 and its taxonomic position is thus illuminated. The remaining fossils are excluded from the Hydrophiloidea due to the absence of any hydrophiloid apomorphy. Hence, our study supports the opinion by Fikáček et al. (2010) that various middle-sized Tertiary beetles were assigned into the hydrophilid genus Hydrobius irrespectively to their real taxonomic position.

## Geology and stratigraphy of the fossil sites

The Tertiary outcrops of Brunstatt and Kleinkems were located in the south-west of central Europe and no longer exist today. Brunstatt was situated south of the city of Mulhouse in France (47°41'N, 7°31'E); Kleinkems was situated in Germany (47°43'N, 7°19'E) northwest of the city of Basel (Switzerland), about 16 km from Brunstatt. Several hundred fossils in total were collected at these localities (Wappler et al. 2005).

According to the reconstruction of the sedimentation history, the limnic sediments of Brunstatt and Kleinkems were deposited on the shore of a very large shallow saline lake (with an area of several hundred square kilometres) with episodical intrusion of fresh water (Lutz 1997). The landscape in this area originated during the formation of the Upper Rhine Graben (URG) which forms the central part of the Cenozoic Central European Rift System. Increased rifting during the late Middle Eocene to Early Oligocene led to the formation of the Mulhouse Potash Salt Basin (also called Potash Basin or Potassic Basin) which is located in the narrowest part of the graben and flanked by the highest of the Vosges Mountains and Black Forest Mountains (Hinsken et al. 2007). A detailed overview of the development of the URG and the Potash Basin is provided by Berger et al. (2005 a, b) and Hinsken et al. (2007).

Lutz (1997) and Mai (1995) assign the age of Brunstatt and Kleinkems to the Lower Oligocene, but Mai (1995) also allocated Brunstatt to the Mammal Reference Level MP20 which corresponds to the Priabonian (37.2–33.9 Ma) in the latest part of the Eocene. Thus, the stratigraphical position of Brunstatt and Kleinkems seems to be close to the Eocene-Oligocene boundary, as is the case of the similar fossil site of Altkirch in France (Wappler et al. 2005). According to Mai (1995) [based on Lakowitz (1895)], the palaeoclimate of Brunstatt was characterised by an average annual temperature of 18°C and abundant rainfall.

It seems very likely that the sediments of Brunstatt and nearby Kleinkems are nearly identical in age and genesis. Lutz (1995) even combined both localities in his study reconstructing their paleoenvironment, and according to Mai (1995), plant fossils from Brunstatt and Kleinkems are both deposited in the same layers of laminated clay (‘plattiger Steinmergel’).

## Material and methods

Only the fossils from the locality of Kleinkems mentioned by Théobald (1937) deposited currently in the Naturhistorisches Museum in Basel, Switzerland (NHMB) were studied for this paper. The material originally examined by Förster (1891) was deposited at the ‘Service de la Carte Geologique de Strasbourg’ (Théobald 1937) and is considered to have been lost or destroyed during World War II on the basis of information we received from Jean Claude Horrenberger (École et Observatoire de la Terre, Strasbourg, France) as well as two letters sent to Volker Püthz, a specialist on Staphylinidae, by Marguerite Wolf (Université Louis Pasteur, Institut de Géologie, Strasbourg, France) in July 1967 and September 1971 (Püthz, pers. comm. 2010). The identity of species missing from the Kleinkems material is only discussed briefly on the basis of the original drawings by Förster (1891).

Fossils were examined using the Olympus SZ61 binocular microscope. Photographs were taken using the Canon MP-E 65 mm macro lens attached to the Canon EOS 550D camera. Drawings were traced from photographs. Abbreviations used in descriptions are: EL – length of the elytron; TL – total length, a single measurement of length from front of head to apex of elytra; TL-h – total length minus head length, length of body from anterior margin of pronotum to apex of elytra; TW – maximum width of body measured at right angles to TL.

Fossils whose family placement and hence also generic placement remains unclear are cited using the original combination of the name, placing the respective genus name in  quotation marks.

## Taxonomy

### Coleoptera: Adephaga

**Family Dytiscidae**

#### Copelatus convexus

(Förster, 1891) comb. n.

Figs 1–4

Escheria convexa Förster 1891: 359, plate XI, Figs 9a,b (original description from Brunstatt); Handlirsch 1908: 767 (catalogue).
Hydrobius convexus : Théobald 1937: 168, plate XII, Fig. 29 (transferred to Hydrobius, recorded from Kleinkems); Hansen (1999: 319, catalogue).

##### WWW site on Wikispecies.

http://species.wikimedia.org/wiki/Copelatus_convexus

##### Material examined.

Neotype, by present designation (NHMB): R91 (imprint) + R74 (counter-imprint) from the locality of Kleinkems (SW Germany, ca. Eocene-Oligocene boundary): fossil of the whole beetle in dorsal view, head, pronotum and elytra almost completely preserved; appendages missing.

##### Redescription.

**Figures 1–6.**
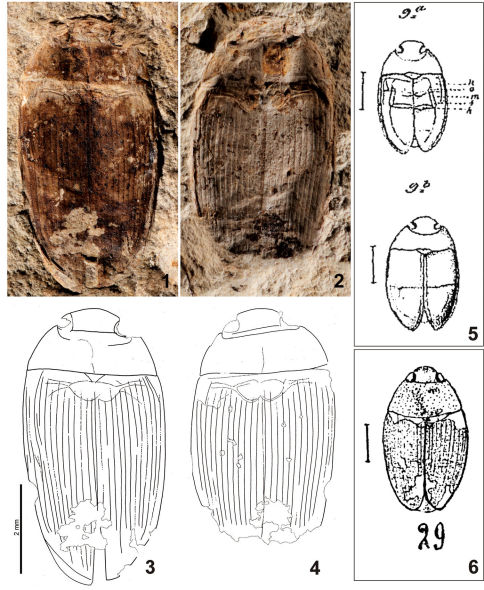
Copelatus convexus (Förster, 1891). **1–4** neotype (**1, 3** NHMB R91; **2, 4** NHMB R74) **5** original illustrations of the holotype by Förster (1891) **6** drawing of the specimen NHMB R91 by Théobald (1937).

Body oblong-oval, broadest in basal third of elytra. Head relatively broad; compound eyes large, not exceeding body outline; clypeus rounded. Pronotum broadest between posterior angles, lateral margins regularly, moderately curved. Mesoscutellar shield well preserved, broadly triangular. Base of elytra as broad as pronotal base; lateral margins of elytra moderately curved. Only mesocoxae, part of metathoracic anepisternum, and probably part of apical abdominal ventrite perceptible from ventral part of body (Figs 1–4).

Surface sculpture. Pronotum with distinct longitudinal median impression, and poorly perceptible short longitudinal striolae on disc. Elytra with 12 moderately impressed longitudinal striae.

Measurements. TL: 6.3 mm, TL-h: 5.6 mm, TW: 3.2 mm. EL: 5.2 mm.

##### Notes on the type material.

Theneotype corresponds well with the original description and drawings by Förster (1891) in the following characters: (i) general body shape; (ii) shape of the pronotum with projecting anterior angles; (iii) elytra with large number of longitudinal striae [preserved only in posterior portion of elytra in the holotype and their number is therefore estimated by Förster (1891) to be at least 10; 12 striae are present in the specimen from Kleinkems]; (iv) shape and proportion of the scutellar shield [much wider than long]; (v) body size [TL = 6.5 mm, EL = 4.3 mm, TW = 3.8 mm for the specimen from Brunstatt according to Förster (1891)]. The body proportions differ slightly between both specimens (the specimen from Brunstatt is relatively wider), but this may easily have been caused by deformation during the fossilization process or by the inaccuracy of the drawings by Förster (1891) which is quite usual for historic authors (e.g., compare the drawings by Théobald (1937) in Figs 6 and 12 with the actual appearance of the respective fossils). Moreover, Förster (1891) mentioned that his fossil resembles the dytiscid genus Agabus in most characters and did not assign it to the Dytiscidae merely because of its ventral morphology which was reconstructed by him as resembling that of Polyphaga (Fig. 5). It seems that Théobald (1937) examined Förster’s types as he mentioned certain details which are absent in Förster’s (1891) original publication, and his opinion about the conspecificity therefore also has to be considered as a strong argument.

The reasons provided above together with the same age, geological origin and geographical proximity of both outcrops (Brunnstatt and Kleinkems) provide strong support for the conspecificity of both specimens mentioned by Théobald (1937). As the specimen from Brunstatt (i.e., the holotype) is lost, we consider it adequate to designate the specimen from Kleinkems as the neotype.

##### Generic attribution.

The preserved characters of the ventral morphology, i.e. the narrow metathoracic anepisternum arising from the median coxal cavity and the anepisterno-metaventral suture directed lateroposteriad correspond closely with the ventral morphology of the Dytiscidae (see, e.g., Fig. 7.6.1 in Balke (2005)). The hydrodynamic body shape, large eyes, broad mesoscutellar shield, medium body size and distinct elytral striae enable us to classify the specimen without any doubt as belonging to the genus Copelatus Erichson, 1832 of the family Dytiscidae.

Copelatus is currently pantropical in its distribution and contains more than 400 described species (Nilsson 2001). Most species of Copelatus are characterised by longitudinal elytral striae whose number has been used to group the species into species groups (Sharp 1882); only a few species have smooth elytra (e.g., Hájek et al. 2010). Although the presence and number of elytral striae provides only limited evidence of phylogeny (Balke et al. 2004), the species groups delimited by number and position of elytral striae are frequently used as a tool for better orientation within the genus (e.g., Guignot 1961; Guéorguiev 1968; Nilsson et al. 1997). The European species previously classified in Copelatus have elytra without striae and have been transferred to the genus Liopterus Dejean,1833 by Balke et al. (2004); they are not closely related to the fossil dealt with in this study.

##### Comparison with other Copelatus species.

Altogether five species of fossil Copelatus species are known: Copelatus aphroditae Balke, 2003 from Baltic amber (Eocene), Copelatus predaveterus Miller, 2003 from Dominican amber (Miocene) (Miller and Balke 2003), and Copelatus fossilis Říha, 1974, Copelatus ponomarenkoi Říha, 1974 and Copelatus stavropolitanus Říha, 1974 from the Miocene deposit of Stavropol (Říha 1974). The differences between all known species are summarized in Table 1.

**Table 1.** List of fossil species of the genus Copelatus, their basic morphological characteristics and their age. Body measurements in italics are estimated from usual TL/EL ratio in Copelatus.

Species	Period	Body length	Number of elytral striae	Species group (Nilsson 2001; Miller and Balke 2003)
Copelatus aphroditae	Eocene	5.0 mm	19 discal	Copelatus aphroditae-group
Copelatus convexus	Eocene- Oligocene boundary	6.3–6.5 mm	12 discal	Copelatus convexus-group
Copelatus fossilis	Miocene	6.1–6.5 mm	10 discal + 1 submarginal	Copelatus erichsoni-group
Copelatus ponomarenkoi	Miocene	5.5–5.6 mm	6 discal + 1 submarginal	Copelatus irinus-group
Copelatus predaveterus	Miocene	3.8–4.4 mm	11 discal + 1 submarginal	Copelatus trilobatus-group
Copelatus stavropolitanus	Miocene	5.1 mm	11 discal	Copelatus nigrolineatus-group

Copelatus convexus differs from all known fossil and extant species of the genus in the presence of 12 longitudinal striae on each elytron. Sharp (1882) erected a group characterized by 12 discal striae (group 7) for a single species Copelatus interruptus Sharp, 1882 which is, however, currently classified in the genus Exocelina Broun, 1886 (Nilsson 2007). In contrast to the fossil Copelatus convexus, this recent species has elytra with numerous short striolae rather than ‘true’ striae, see, e.g. Wewalka et al. (2010). Therefore, Copelatus convexus might be provisionally classified in a separate Copelatus convexus-group. However, it is necessary to point out that the counting of the precise number of lateral elytral striae is problematic in compressed fossils as the imprint of the submarginal stria may coincide with the lateral margin of the body or with the epipleuron. Therefore, we cannot rule out that a short submarginal stria was present in Copelatus convexus although it is not perceptible in the fossil. In this case, Copelatus convexus would belong to the Copelatus simoni-group sensu Nilsson (2001).

### Coleoptera: Polyphaga

**Family *incertae sedis***

#### 'Escheria' crassipunctata

Förster, 1891

Fig. 7

Escheria crassipunctata Förster 1891: 364, plate XI, Fig. 11 (original description from Brunstatt); Handlirsch 1908: 767 (catalogue).
Hydrobius crassipunctatus : Théobald 1937: 169, plate II, Fig. 28 (transferred to Hydrobius, referred from Kleinkems); Hansen 1999: 319 (catalogue).

##### Taxonomic notes.

As in the case of Copelatus convexus, Théobald (1937) transferred Escheria crassipunctata to the hydrophilid genus Hydrobius and assigned fossil specimen no. R 707 from the locality of Kleinkems (deposited in NHMB) to this species. We have examined the specimen from Kleinkems for this study (Figs 10–11) but we cannot confirm that it is conspecific with Escheria crassipunctata for the following reasons: (i) the elytra are slightly constricted sub-basally in the specimen from Kleinkems, but evenly rounded laterally in Escheria crassipunctata; (ii) the body outline is distinctly interrupted between the pronotum and the elytra, but it is uninterrupted in Escheria crassipunctata, (iii) eyes are large and globular in the specimen from Kleinkems, but relatively small in Escheria crassipunctata. A more detailed comparison is impossible as the holotype of Escheria crassipunctata is lost and was moreover preserved in dorsal view based on the drawing by Förster (1891), whereas the specimen from Kleinkems is preserved in ventral view.

Based on the original drawing by Förster (1891), Escheria crassipunctata does not bear any synapomorphy of the Hydrophiloidea. For this reason, the species is removed from the fossil record of the Hydrophiloidea and is placed in Polyphaga *incertae sedis*.

Specimen no. R707 from Kleinkems does not bear any synapomorphy of the Hydrophiloidea, and moreover bears a combination of characters which excludes its assignment to the Hydrophiloidea: (i) prosternal process wide, (ii) mesocoxal cavities rather wide apart, and (iii) eyes large and globular. The preserved characters of this specimen do not allow an unambiguous family assignment (see Lawrence et al. 1999).

**Figures 7–12.**
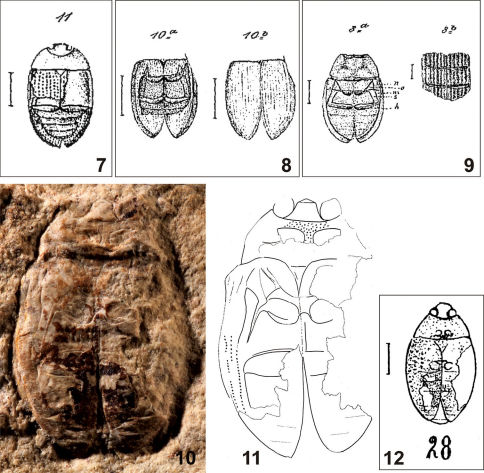
**7** Escheria crassipunctata Förster, 1891, original illustration of the holotype **8** Escheria dimidiata Förster, 1891, original illustration of the holotype **9** Escheria punctulata Förster, 1891, original illustration of the holotype **10–12** specimen NHMB R707 (**10** photograph **11** drawing **12** original drawing by Théobald (1937)).

#### 'Escheria' dimidiata

Förster, 1891

Fig. 8

Escheria dimidiata Förster 1891: 363, plate XI, Figs 10a,b (original description from Brunstatt); Handlirsch 1908: 767 (catalogue)
Hydrobius dimidiatus : Théobald 1937: 169 (transferred to Hydrobius); Hansen 1999: 319 (catalogue).

##### Taxonomic note.

Based on the original drawing by Förster (1891), the morphology of Escheria dimidiata agrees with that of Hydrophilidae: Hydrophilinae in many aspects: (i) mesocoxal cavities transverse, narrowly isolated from each other, (ii) metanepisternum rather narrow, (iii) epipleuron narrow but reaching elytral apex, and (iv) elytron with 10 longitudinal punctural series. None of these characters or their combination is, however, unique for the Hydrophiloidea and may be found in other beetle families as well (see e.g. Lawrence et al. 1999). Moreover, the medium body size (EL: 9 mm according to Förster (1891)) would indicate that the fossil should belong to the subtribes Hydrobiusina or Hydrophilina, whose representatives are characterized by a relatively large and well developed triangular mesoscutellar shield; in contrast, the scutellar shield is very small or reduced in Escheria dimidiata. Moreover, the re-examination of the fossil is impossible as the holotype was lost or destroyed. For all these reasons, Escheria dimidiata is removed from the fossil record of the Hydrophiloidea and is placed in Polyphaga *incertae sedis*.

#### 'Escheria' punctulata

Förster, 1891

Fig. 9

Escheria punctulata Förster 1891: 361; plate XI, Figs 8a,b (original description from Brunstatt); Handlirsch 1908: 767 (catalogue).
Hydrobius punctulatus : Théobald 1937: 169 (transferred to Hydrobius); Hansen 1999: 319 (catalogue).

##### Taxonomic note.

Based on the drawing by Förster (1891), the ventral morphology of this species agrees with that of Hydrophilidae: Hydrophilinae in many characters: (i) mesocoxae transverse and very narrowly separated, (ii) mesepimeron well separated, triangular, (iii) metanepisternum rather narrow; (iv) abdomen with five ventrites. However, none of these characters or their combination is unique for the Hydrophiloidea and may be found in other beetle families as well (see Lawrence et al. 1999). Moreover, two characters illustrated on the drawing and/or mentioned in the original description exclude the placement of Escheria punctulata in the Hydrophiloidea: (i) elytra bear only 6 deeply impressed striae [9–11 striae are present in all Hydrophiloidea with striate elytra, only rarely is the number of series higher but in such cases they are never impressed to striae]; (ii) mesoventrite fused with mesepisternal (i.e. not separated from them by sutures) [in Hydrophiloidea, the mesoventrite is fused to mesepisterna only in derived groups of the Sphaeridiinae which are characterized by a highly elevated median portion of the mesoventrite; the elevated median elevation is missing from the fossil]. For these reasons, Escheria punctulata is removed from the fossil record of the Hydrophiloidea and is placed into Polyphaga *incertae sedis*; its family placement remains unclear.

## Supplementary Material

XML Treatment for Copelatus convexus

XML Treatment for 'Escheria' crassipunctata

XML Treatment for 'Escheria' dimidiata

XML Treatment for 'Escheria' punctulata
